# Effect of Hf-doping on electrochemical performance of anatase TiO_2_ as an anode material for lithium storage

**DOI:** 10.1098/rsos.171811

**Published:** 2018-06-06

**Authors:** Sergey V. Gnedenkov, Sergey L. Sinebryukhov, Veniamin V. Zheleznov, Denis P. Opra, Elena I. Voit, Evgeny B. Modin, Alexander A. Sokolov, Alexander Yu. Ustinov, Valentin I. Sergienko

**Affiliations:** 1 Institute of Chemistry, Far Eastern Branch of Russian Academy of Sciences, Vladivostok 690022, Russia; 2 Far Eastern Federal University, Vladivostok 690950, Russia; 3 National Research Centre ‘Kurchatov Institute’, Moscow 123182, Russia

**Keywords:** Li-ion battery, anatase TiO_2_, doping, anode, nanostructured material, sol–gel process

## Abstract

Hafnium-doped titania (Hf/Ti = 0.01; 0.03; 0.05) had been facilely synthesized via a template sol–gel method on carbon fibre. Physico-chemical properties of the as-synthesized materials were characterized by X-ray diffraction, Raman spectroscopy, scanning electron microscopy, energy-dispersive X-ray analysis, scanning transmission electron microscopy, X-ray photoelectron spectroscopy, thermogravimetry analysis and Brunauer–Emmett–Teller measurements. It was confirmed that Hf^4+^ substitute in the Ti^4+^ sites, forming Ti_1–*x*_Hf*_x_*O_2_ (*x* = 0.01; 0.03; 0.05) solid solutions with an anatase crystal structure. The Ti_1–*x*_Hf*_x_*O_2_ materials are hollow microtubes (length of 10–100 µm, outer diameter of 1–5 µm) composed of nanoparticles (average size of 15–20 nm) with a surface area of 80–90 m^2^ g^–1^ and pore volume of 0.294–0.372 cm^3^ g^–1^. The effect of Hf ion incorporation on the electrochemical behaviour of anatase TiO_2_ in the Li-ion battery anode was investigated by galvanostatic charge/discharge and electrochemical impedance spectroscopy. It was established that Ti_0.95_Hf_0.05_O_2_ shows significantly higher reversibility (154.2 mAh g^–1^) after 35-fold cycling at a *C*/10 rate in comparison with undoped titania (55.9 mAh g^–1^). The better performance offered by Hf^4+^ substitution of the Ti^4+^ into anatase TiO_2_ mainly results from a more open crystal structure, which has been achieved via the difference in ionic radius values of Ti^4+^ (0.604 Å) and Hf^4+^ (0.71 Å). The obtained results are in good accord with those for anatase TiO_2_ doped with Zr^4+^ (0.72 Å), published earlier. Furthermore, improved electrical conductivity of Hf-doped anatase TiO_2_ materials owing to charge redistribution in the lattice and enhanced interfacial lithium storage owing to increased surface area directly depending on the Hf/Ti atomic ratio have a beneficial effect on electrochemical properties.

## 1. Introduction

Nowadays, rechargeable Li-ion batteries (LIBs) are used worldwide as power sources for portable electronics, operating tools, and implantable devices because of their excellence in terms of energy density, cycle life and reliability [1,2]. By contrast, the extensive application of LIBs for hybrid and electric vehicles, uninterruptible power supplies, unmanned underwater vehicles, renewable alternative energy systems is limited owing to a number of issues, foremost being power density and safety. The overwhelming majority of LIBs consist of a lithiated metal oxide cathode (e.g. LiCoO_2_, LiNiO_2_, LiMnO_2_ and LiFePO_4_) and a carbonaceous anode (usually graphitized carbon or graphite). Owing to strong oxidizing and reducing agents used as electrode-active materials, the LIBs’ operating voltage is high, e.g. 3.6–3.7 V for a Li*_x_*C_6_/Li_1–*x*_CoO_2_ system. At the same time, because of the Li*_x_*C_6_ potential being close to that of Li/Li^+^, the electrolyte undergoes reduction that leads to blocking of solid electrolyte interphase (SEI) formation on the anode surface. The latter results in both irreversible capacity loss and, more unfavourably, tree-like lithium dendrite growth (especially intensive at high *C*-rates) that has a negative effect on LIB safety [3,4]. In this case, the safety of LIBs is sufficient for traditional applications, whereas it is an insurmountable obstacle for medium- and large-scale energy storage requiring faster charge/discharge.

Titania polymorphs, mainly anatase and TiO_2_(B), are more suitable anode materials for high power density, high-safety LIBs owing to their higher Li^+^ insertion potentials (from 1.5 to 1.8 V) when compared with graphite (lower than 0.3 V). It is well known that, at the potential higher than 1.2 V (cathodic limit of the electrochemical window for the typical LIB electrolyte solution), the formation of SEI on the anode surface can be effectively avoided [5,6]. It may prevent lithium dendrite growth and improve greatly the LIB's power density and safety. Furthermore, anatase TiO_2_ has a better stability during cycling because of smaller volume changes (lower than 4%) in comparison with graphite (9–10%). On the other hand, because of a higher theoretical-specific capacity of 335 mAh g^–1^ titania possesses almost twice the energy density in comparison with Li_4_Ti_5_O_12_ that recently has received increasing interest for similar reasons [7]. The last but not least is the natural abundance, low cost (at least a third of Li_4_Ti_5_O_12_) and environmental friendliness of TiO_2_. Unfortunately, anatase TiO_2_ possesses slow diffusivity of Li^+^ ions (10^–17^–10^–9^ cm^2^ s^–1^) into the crystal lattice that hampers its application in the LIB anode [8]. Moreover, titania is a semiconductor-type material, possessing a wide band gap (3.2 eV for anatase) that results in poor conductivity (10^–12^–10^–7^ S cm^–1^) and additionally limited electrochemical performance of the LIB [9].

It was established elsewhere [10,11] that nanostructuring improves significantly the Li-storage properties of titania, e.g. facilitates the Li^+^ ion diffusion and intensifies the redox electrochemical reactions. However, as it turned out, the particles’ nanoscale size is not sufficient to design the anatase TiO_2_ suitable for commercialization in the LIB anode. A variety of methods has been proposed to improve the electrochemical performance of anatase TiO_2_, e.g. interconnection of titania with carbonaceous materials (single-wall carbon nanohorns [12], carbon nanofibres [13], graphene [14]) or conducting polymers (polyaniline [15], polypyrrole [16]), core-shell structures (MoS_2_/TiO_2_ [17], Sn/TiO_2_ [18], MnO*_x_*/TiO_2_ [19]). At the same time, it is difficult to provide reliable charge carrier transport pathways owing to illusive uniformity of composite materials and/or nanoparticle agglomeration. Additionally, most of the approaches are unprofitable, inconvenient and include a number of sophisticated stages.

Recently, substitutional metal ion (M*^n^*^+^) doping of anatase TiO_2_ has attracted great attention as a promising way to improve its electrochemical performance [20–23]. It is well known that incorporation of M*^n^*^+^ ions with an oxidation number more than 4+ (e.g. V^5+^ [24] or Mo^6+^ [25]) causes charge redistribution owing to reduction of Ti^4+^ to Ti^3+^ that enhances electronic conductivity of titania, whereas partial substitution of Ti^4+^ into the anatase TiO_2_ lattice by M*^n^*^+^ ions with an oxidation number of less than 4+ (e.g. Ni^2+^ [26] or Fe^3+^[27]) creates an oxygen vacancy that additionally increases ionic conductivity of TiO_2_. On the other hand, as it was noted, in our earlier work [28], the ionic radius of the dopant is no less important in terms of anatase TiO_2_ electrochemical behaviour. In particular, differences in the ionic radius values of Ti^4+^ and M*^n^*^+^ leads to changing crystal lattice parameters of TiO_2_ after doping that may result in the facilitation or slowing of Li^+^ ions’ diffusion kinetics. Thus, in order to design an efficient anatase TiO_2_ anode for high power density, high-safety LIBs, the balancing between the ionic radius and the oxidation number of the dopant is a key factor. Hence, a clear understanding of the doping strategy from the point of view of the M*^n^*^+^ ionic radius is strongly required.

Here, the doping with Hf^4+^ of nanostructured anatase TiO_2_ tubes by an inexpensive template sol– gel method is reported. The relationship between the electrochemical behaviour of Ti_1–*x*_Hf*_x_*O_2_ (*x* = 0.01; 0.03; 0.05) in the LIB anode and the ionic radius of a substitutional agent is investigated by charge/discharge tests and electrochemical impedance spectroscopy. Based on the results in this work coupled with the data published in our previous report [28], the importance of the dopant ionic radius is discussed in detail and a good grasp of the principles of TiO_2_ doping is achieved.

## 2. Experimental section

### 2.1. Synthesis procedure

Hf-doped anatase TiO_2_ in the form of nanoparticle-structured tubes was synthesized by a template sol–gel procedure, which had been developed by us earlier [29]. Analytical grade titanium tetrachloride (Component-Reaktiv, Russia) and hafnium oxychloride hydrate (Sigma-Aldrich, USA) were used as precursors without any purification. As a template, Busofit-T055 carbon fibre (Khimvolokno, Belarus) was applied. As the Busofit-T055 fibre contains approximately 0.01% silicon as an impurity, the preliminary autoclave treatment with NH_4_HF_2_ at 130°C was carried out. As a result, silicon concentration decreases by 30 times. In a typical synthesis process, 0.5 ml TiCl_4_ was added to 1 l distilled water under vigorous stirring until full dissolution. Subsequently, HfOCl_2_·8H_2_O was placed into the obtained solution with the Hf/Ti atomic ratios of 0.01 (Ti_0.99_Hf_0.01_O_2_), 0.03 (Ti_0.97_Hf_0.03_O_2_) and 0.05 (Ti_0.95_Hf_0.05_O_2_). After that, prepared solution was deposited on the surface of the treated Busofit-T055 carbon fibre template. Finally, slow annealing at 500°C under air for 2 h was performed for template removal. The undoped TiO_2_ was synthesized under the same conditions without the HfOCl_2_·8H_2_O for comparison.

### 2.2. Characterization

The particles’ surface morphology was observed by scanning electron microscopy (SEM) on a S5500 microscope (Hitachi, Japan) The samples for SEM were prepared by spreading the undoped or Hf-doped anatase TiO_2_ on sticky conductive adhesive tape. Additionally, the microstructure was investigated by transmission electron microscopy (TEM) on a Titan 80–300 (FEI, USA) equipped with a spherical aberration (Cs) corrector of an electron probe. The TEM was operated under an acceleration voltage of 300 kV in a bright-field imaging and high-angular dark field scanning (HAADF STEM) mode. For (S)TEM observations, the specimens were dispersed during 5 min in the ultrasonic bath with ultrapure water, which was obtained using a Milli-Qwater purification system (Millipore, USA). Then, a drop of suspension was applied to a copper grid with a lacy carbon support film. TEM samples were treated for 20 s in a Model 1020 plasma cleaner (Fischione, USA) using the Ar/O_2_ gas mixture to reduce carbohydrate contamination. The Gatan Digital micrograph software was used for image processing and analysis. STEM image simulation was performed with the JEMS electron microscopy software. Energy-dispersive X-ray (EDX) microanalysis was performed on the Versa 3D SEM (FEI, USA) equipped with the high-count rate silicon drift detector Octane-plus (EDAX, USA). The accelerating voltage for EDX mapping was set to 15 keV and the beam current was 1.7 nA. The specific surface area was determined using the ASAP 2020 V3.04 H (Micrometrics, USA) spectrometer from isotherms of low-temperature adsorption of nitrogen by the Brunauer–Emmett–Teller (BET) method. The pore diameter and total volume of pores were evaluated using the original density functional theory. The surface chemistry was revealed by X-ray photoelectron spectroscopy (XPS) on a Phoibos 150 hemispherical electrostatic energy analyser (SPECS, Germany). Mg K*_α_*-radiation was used as the primary excitation source. The measurements were refined for possible charging effects by assigning a value of 285.0 eV to the C 1 s reference line resulting from the thin layer of residual hydrocarbon. To investigate the crystal structure of materials, X-ray diffraction (XRD) and Raman spectroscopy were used. The D8-Advance diffractometer (Bruker, Germany) with *Cu K_α_*-radiation was applied for XRD measurements. Identification of the XRD data was performed using the EVA program with the PDF-2 (2006) powder database. Raman studies were conducted on the RFS-100/S spectrometer (Bruker, Germany) equipped with a Ge detector. As the excitation source an Nd:YAG laser with a wavelength of 1064 nm was applied. Thermogravimetry (TG) was carried out on the DTG-60H derivatograph (Shimadzu, Japan) at a heating rate of 5°C min^–1^ under an air atmosphere from room temperature to 1000°C.

### 2.3. Electrochemical tests

The working electrode was composed of anatase Ti_1–*x*_Hf*_x_*O_2_ (*x* = 0; 0.01; 0.03; 0.05) as an active material, Super P carbon black (Alfa Aesar, USA) as a conductive additive and polyvinylidene fluoride (MTI, USA) as a binder at a weight ratio of 80 : 10 : 10. The mixture was homogenized in the *N*-methylpyrrolidone solvent (Ekos-I, Russia) using the C-MAG HS 7 magnetic stirrer (IKA, China) at a rate of 300 r.p.m. for 15 h to prepare a homogeneous viscous slurry. Then, the electrode slurry was spread onto a copper current collector sheet (thickness is 11 µm) by the doctor blade method using the AFA-I instrument (MTI, USA). The electrode sheet was dried at 70°C for 7 h in the DZF-6020-110P oven (MTI, USA). The T06 tool (MTI, USA) was used for the cutting of a round electrode disc (diameter is 1.5 cm) from the sheet. Finally, the working electrode was compacted at 1000 kg cm^–2^ on a C3851 press (Carver, USA) and dried under vacuum at 110°C overnight. The mass loading of active material was approximately 2 mg cm^–2^.

The half-cell was assembled in a 890-NB glove box (Plas-Labs, USA) under a dry (H_2_O < 1 ppm) Ar-filled (purity is 99.999%) atmosphere. A two-electrode ECC-STD cell (Bio-Logic, USA) was applied to test the electrochemical performance. Lithium metal (Lithium-Element, Russia) was used as a counter and reference electrode. A 1 M solution of LiClO_4_ in the mix of propylene carbonate and dimethoxyethane at a volume ratio of 5 : 1 (Ekotech, Russia) was applied as the electrolyte. To prevent short circuit, a Celgard 2400 polypropylene separator (Celgard, USA) was used.

Galvanostatic cycling tests at the rates of *C*/10 and 1*C* (*C* is equal to 335 mA g^–1^) were performed on a 1470E potentiostat/galvanostat (Solartron, UK) between the potentials of 1.0 and 3.0 V. In the experiments, because of applying half-cells, the discharge implies a lithiation process, while the charge implies de-lithiation. Electrochemical impedance spectroscopy (EIS) data were collected on a 1455 (Solartron, UK) frequency response analyser at room temperature for the fresh cells at open-circuit potential with an AC amplitude of 5 mV over a frequency range from 1 MHz to 100 mHz. The measurements were carried out on at least six half-cells for each test.

## 3. Results and discussion

### 3.1. Morphology, composition and crystal structure of Ti_1–*x*_Hf*_x_*O_2_

**Figure 1.**
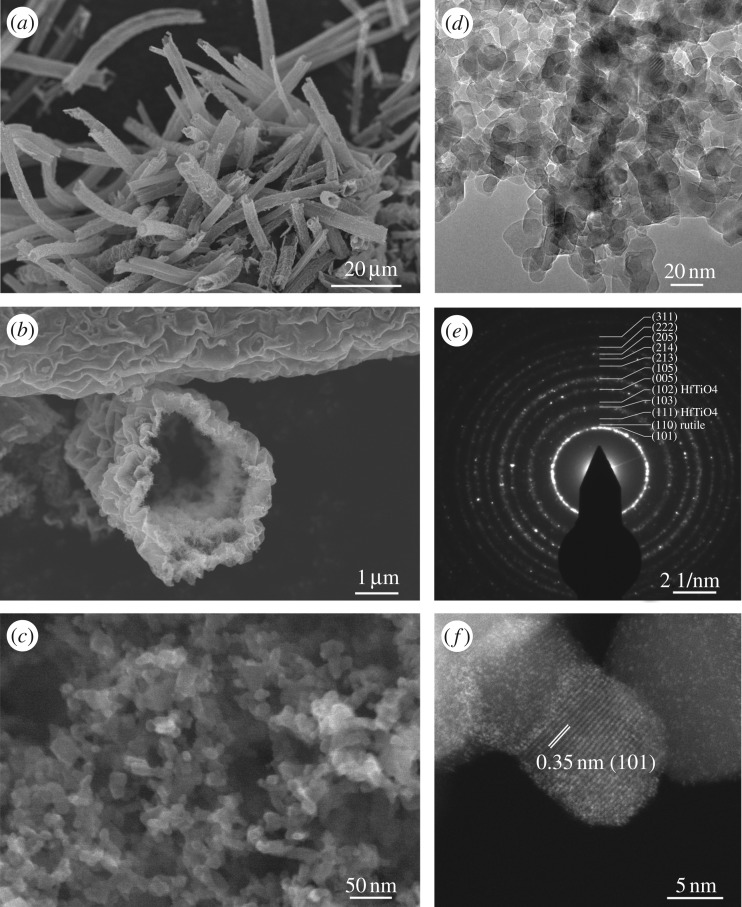
(*a*–*c*) SEM micrographs, (*d*) TEM image in the bright-field mode, (*e*) corresponding SAED pattern, and (*f*) HAADF STEM picture for the Ti_0.95_Hf_0.05_O_2_ material.

The SEM investigations show that as-synthesized undoped TiO_2_, Ti_0.99_Hf_0.01_O_2_, Ti_0.97_Hf_0.03_O_2_ and Ti_0.95_Hf_0.05_O_2_ samples have a similar microstructure. In this way, the Ti_0.95_Hf_0.05_O_2_ micrographs are presented as an example. Hence, the anatase TiO_2_-based materials consisted of tubes ranging in length from 5 to 50 µm (figure 1*a*). At the same time, some amounts of tubes with lengths up to 300 µm were observed. The outer diameter of tubes varies in the range 2–5 µm (figure 1*b*). Tubes have a nanostructured surface, their walls composed of nanoparticles (figure 1*c*). For a profound insight into the nanoparticulate morphology of anatase TiO_2_ doped with Hf^4+^, the (S)TEM investigations of Ti_0.95_Hf_0.05_O_2_ have been carried out. From the TEM images it can be seen that tubes consist of close-to-spherical nanoparticles with an average size of 15–20 nm (figure 1*d*). Additionally, TEM analysis demonstrates the structure of Ti_0.95_Hf_0.05_O_2_ presented by nanoparticles with inter-particle pores. The N_2_ adsorption isotherm and the corresponding evaluation of pore diameter and total volume of pores further confirm the porous microstructure of the Hf-doped TiO_2_. The average pore diameter for undoped titania is 1.48 nm and the total pore volume is 0.294 cm^3^ g^–1^. Ti_0.95_Hf_0.05_O_2_ possesses a pore diameter of 3.17 nm and total pore volume of 0.372 cm^3^ g^–1^. Furthermore, the Hf^4+^ doping of anatase titania increases the BET surface area from 80 m^2^ g^–1^ (undoped TiO_2_) to 90 m^2^ g^–1^ (Ti_0.95_Hf_0.05_O_2_), which seems due to the slightly reduced particle size. According to the literature [30], porosity and surface area have a beneficial effect on the electrochemical activity of anatase TiO_2_.

The selective area electron diffraction (SAED) pattern (figure 1*e*) highlights the anatase TiO_2_ structure of nanoparticles: (101), (103), (005), (105), (213), (214), (205), (222) and (311) planes. The appearance of spots with inter-planar spacing of 0.32 nm associated with the (110) rutile plane indicates the existence of a rutile phase as an impurity. Spots with low intensity that could be measured as inter-planar spacings of 2.89 and 2.22 nm in the SAED pattern could be related to the (111) and (102) planes of HfTiO_4_ traces, and indicate that the maximum concentration of Hf into the TiO_2_ lattice is achieved for Ti_0.95_Hf_0.05_O_2_. Hence, the further increase of the Hf/Ti ratio (more than 0.05) is not rational.

**Figure 2.**
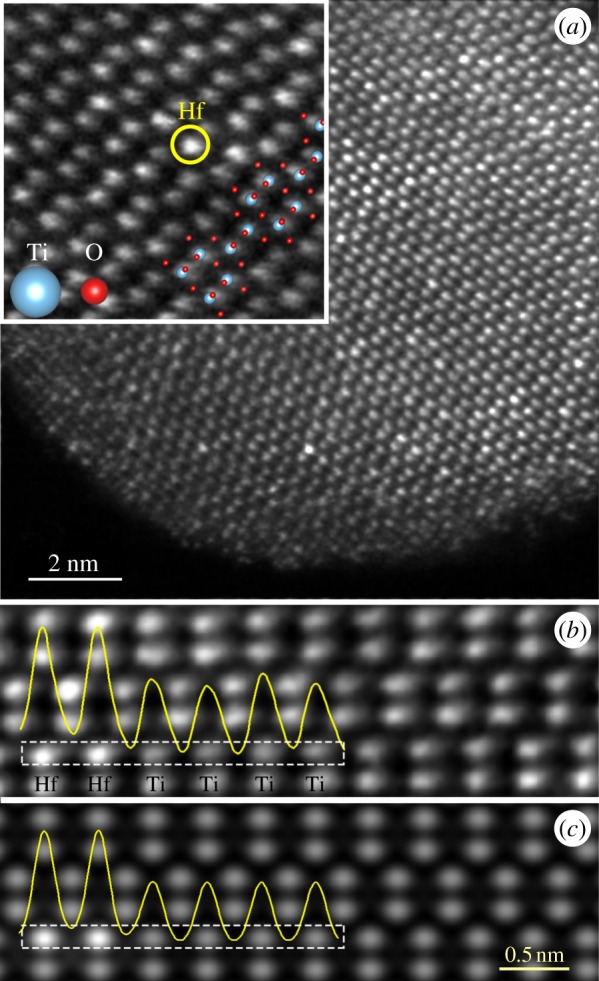
(*a*) *Z*-contrast image of Ti_0.95_Hf_0.05_O_2_ in the (100) orientation with a higher magnification picture (inset) of one individual dislocation core, (*b*) experimental, and (*c*) simulated crystal structure of anatase TiO_2_. The bright spots represent the Ti and Hf columns; pure O columns are not visible.

From the HAADF STEM image of the Ti_0.95_Hf_0.05_O_2_ sample (figure 1*f*), showing TiO_2_ nanoparticles in different crystallographic orientation, one can see the inter-planar spacing of 0.35 nm assigned to the (101) plane of anatase TiO_2_. As is well known, the imaging in the HAADF STEM mode is atomic number-sensitive, so the contrast is proportional to *Z*^2^. Hence, owing to a large difference in atomic numbers (*Z*_Hf_ = 72 versus *Z*_Ti_ = 22), Hf atoms appear much brighter than the Ti species. Indeed, STEM imaging of Ti_0.95_Hf_0.05_O_2_ in the *Z*-contrast mode (figure 2*a*) reveals that, in some cases, Hf atoms occupy the positions of titanium in the anatase TiO_2_ crystal lattice. One of those Hf atoms is marked with a yellow circle in figure 2*a*, inset with higher magnification. Simulations of HAADF STEM images were performed to compare the difference in contrast presented at experimental images. The parameters of the microscope were set close to experimental ones. Two single Hf atoms were placed in the titanium atoms’ positions. Figure 2*b* and *c,* respectively, represents an experimental and simulated structure of anatase TiO_2_ in the (100) orientation, where the unit cell *c* direction aligned vertically. For qualitative interpretation, the intensity line profiles are shown in both images. It can be concluded that simulated data are in a good agreement with experimental ones and it is obvious that Hf atoms are incorporated into the lattice in the position of titanium atoms. In this study, we did not aim to visualize the oxygen atoms in anatase TiO_2_ structure, as it was previously reported [31]. Moreover, in the case of the nanoparticles, it is rather difficult to obtain exactly the predetermined zone axis, and also the weak scattering intensity of oxygen atoms makes them invisible in the presented micrographs.

According to the elemental mapping, Ti, Hf and O elements (electronic supplementary material, figure S1) are uniformly distributed in Ti_0.95_Hf_0.05_O_2_. Thus, Hf is incorporated homogeneously into the anatase TiO_2_ crystal lattice. Moreover, EDX measurements give the Hf/Ti atomic ratio to be equal to 0.047, which is close to the aspired ratio of 0.05. Additionally, Si traces of 1.8 ± 0.2 at.% that originated from the Busofit-T055 fibre template were detected as an impurity for all TiO_2_ samples. In view of the atomic concentrations of Ti, Hf, Si and O, the presence of silicium in the form of SiO_2_ can be assumed.

**Figure 3.**
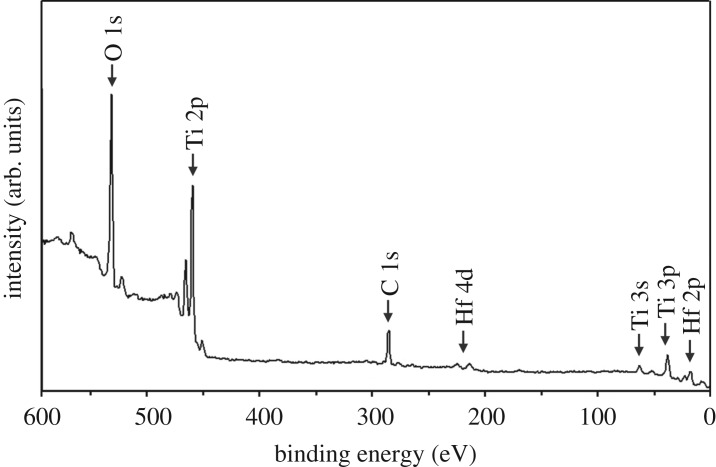
XPS survey scan for the Ti_0.95_Hf_0.05_O_2_ sample.

To examine the oxidation state of the Hf dopant, XPS analysis was carried out for the Ti_0.95_Hf_0.05_O_2_ sample. The XPS survey spectrum (figure 3) shows that Ti, O, Hf and C elements are clearly detected in the material, which is in accordance with EDX data. By contrast, Si was not found, which seems to be owing to a lower penetration depth (30 Å) of the XPS method in comparison with EDX (approx. 1 µm). The high-resolution spectrum of Ti 2p (electronic supplementary material, figure S2a) shows two peaks of Ti 2p3/2 at 459.1 eV and Ti 2p1/2 at 464.8 eV, indicating that titanium is present as Ti^4+^ [32]. Note that, for Hf-doped TiO_2_, there are no signals of Ti^3+^ (approx. 455.0 eV ^27^) in the Ti 2p spectrum results. This seems to indicate that the Ti oxidation state has not been changed owing to Hf doping. The O 1 s peak (electronic supplementary material, figure S2b) exhibits two main contributions. In particular, the binding energy of 530.6 eV belongs to oxygen atoms of the TiO_2_ lattice [33], whereas the band at 532.5 eV corresponds to chemisorbed water, and C─O and O═C─O bonds [34]. From the XPS results (electronic supplementary material, table S1), note that the O/Ti atomic ratio is close to 2 (46.0/20.5). The Hf 4f7/2 and Hf 4f5/2 peaks located at 16.8 eV and 18.4 eV (electronic supplementary material, figure S2c), respectively, indicate that the Hf oxidation state in the as-synthesized Hf-doped anatase TiO_2_ is 4+ [35]. The Hf/Ti atomic ratio in Ti_0.95_Hf_0.05_O_2_ was estimated to be 0.054 (electronic supplementary material, table S1), which is in agreement with EDX measurements and the synthesis procedure. The XPS data processing shows the multicomponent character of the high-resolution C 1 s spectrum (electronic supplementary material, figure S2d). The binding energies of 287.3 and 289.5 eV are associated with the C─O and O═C─O groups [34], respectively, while the value of 285.0 eV was related to the C─C or/and C─H bonds [36].

**Figure 4.**
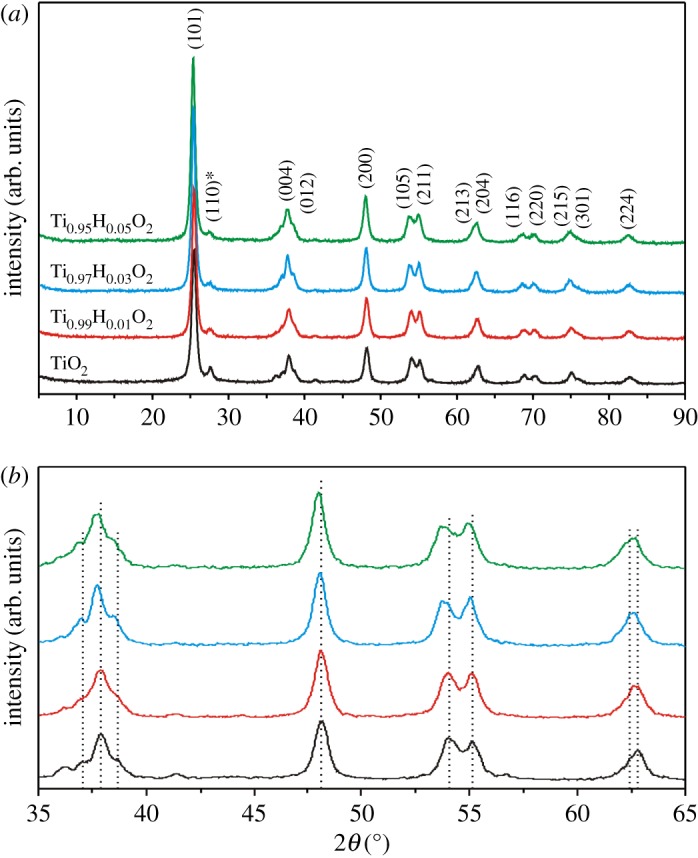
(*a*) XRD patterns for the Ti_1–*x*_Hf*_x_*O_2_ samples with different Hf/Ti ratios from 0 to 0.05 and (*b*) magnified picture between 2*θ* values of 35° and 65°, obviously representing the peak shifts.

Figure 4 shows the XRD patterns of undoped anatase TiO_2_ as well as of samples with different Hf-doping levels. The as-synthesized TiO_2_-based materials are well ascribed to the (101), (003), (004), (012), (200), (105), (211), (213), (204), (116), (220), (215), (301) and (224) reflections of the anatase phase with a tetragonal structure (JSCD no. 00-021-1272, space group *I*4_1_/*amd* (figure 4*a*)). All the diffraction peaks are sharp, indicating favourable crystallinity. As expected, it is observed that the XRD peaks shift with increase in Hf concentration (figure 4*b*). This phenomenon indicates changing anatase TiO_2_ unit cell parameters. The ionic radius of Hf^4+^ is equal to 0.71 Å (CN = 6), while the radius of Ti^4+^ is equal to 0.604 Å (CN = 6) [37]. In accordance with the DFT model of Koudriachova *et al*. [38], it can be assumed that the Hf^4+^ dopant substitutes the Ti^4+^ ions in the crystal lattice of the TiO_2_ host and retains the original coordination number. As a result of Hf^4+^ ion incorporation into the anatase TiO_2_ structure, the difference in the ionic radius of metal ions increases lattice parameters. Indeed, the analysis of XRD patterns of undoped TiO_2_, Ti_0.99_Hf_0.01_O_2_, Ti_0.97_Hf_0.03_O_2_ and Ti_0.95_Hf_0.05_O_2_ samples shows that doping with Hf^4+^ results in increase in the *a* and *с* lattice constants of the anatase TiO_2_ unit cell. This confirms that metal ion substitution in the Ti^4+^ sites forms anatase Ti_1–*x*_Hf*_x_*O_2_ (*x* = 0.01; 0.03; 0.05) solid solutions. The unit cell volume (*V*) of Ti_1–*x*_Hf*_x_*O_2_ increases in the range 136.914–138.062 Å^3^ (table 1), directly depending on the Hf/Ti atomic ratio. As reported previously [26], the latter is favourable for reversible Li^+^ ion intercalation. From the XRD patterns there are no peaks attributed to the HfTiO_4_ phase, which clearly confirms its trace quantity. The low-intensity peak at 27.4° (figure 4*a*, asterisk) corresponds to (110) reflection of the rutile phase (JSCD no. 00-021-1276, space group *P*4_2_/*mnm*), which coexisted with the anatase as the main phase.

**Table 1.** Changing *a* and *с* lattice constants and unit cell volume *V* of undoped and Hf-doped anatase TiO_2_.

sample	*a* (Å)	*c* (Å)	*V* (Å^3^)
TiO_2_	3.785	9.514	136.914
Ti_0.99_Hf_0.01_O_2_	3.792	9.520	136.891
Ti_0.97_Hf_0.03_O_2_	3.798	9.550	137.796
Ti_0.95_Hf_0.05_O_2_	3.801	9.556	138.062

**Figure 5.**
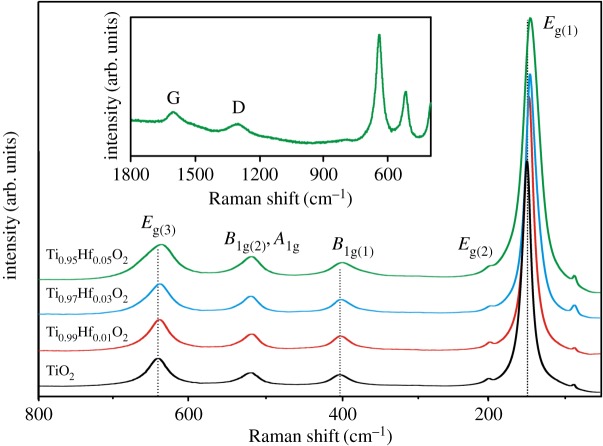
Raman spectra for undoped TiO_2_, Ti_0.99_Hf_0.01_O_2_, Ti_0.97_Hf_0.03_O_2_ and Ti_0.95_Hf_0.05_O_2_ materials. The inset displays the D and G bands in the Raman spectrum for Ti_0.95_Hf_0.05_O_2_.

The Raman spectra (figure 5) of as-synthesized undoped TiO_2_, Ti_0.99_Hf_0.01_O_2_, Ti_0.97_Hf_0.03_O_2_ and Ti_0.95_Hf_0.05_O_2_ correspond to the crystallization of nanoparticles with anatase crystal structure. The results confirmed that incorporation of Hf^4+^ ions has a significant influence on the TiO_2_ unit cell. Indeed, for Hf-doped anatase TiO_2_ the shift of *E*_g(1)_, *B*_1 g(1)_ and *E*_g(3)_ peaks to lower frequencies was found (electronic supplementary material, table S2). The shift of the *E*_g(3)_ peak from 638.7 to 636.1 cm^–1^ in the Raman spectra of Ti_1–*x*_Hf*_x_*O_2_ (*x* = 0; 0.01; 0.03; 0.05) corresponds to the stretching vibrations associated with a weakening of Ti─O bonds, confirming the fact that Hf^4+^ is incorporated as a substitutional dopant. The main contribution to the energy of the *B*_1 g(1)_ deformation mode originates from Ti^4+^ ions. Hence, because of Hf^4+^ substitution in the Ti^4+^ sites, the average distance between ions increased, which results in the low-frequency shift (between 396.9 and 395.1 cm^–1^) of *B*_1 g(1)_. The high-intensity *E*_g(1)_ peak undergoes the greatest shift (from 147.5 to 143.4 cm^–1^) because of its sensitivity to changes in unit–cell parameters of TiO_2_ owing to the incorporation of Hf^4+^ ions. Additionally, the shift of *E*_g(1)_ may also indicate the existence of oxygen vacancies caused by increasing *a* and *c* lattice parameters. The appearance of oxygen vacancies in TiO_2_ results in reduction of some Ti^4+^ to Ti^3+^ in order to maintain the charge balance [23]. However, no Ti^3+^ signal has been detected from XPS data for Ti_0.95_Hf_0.05_O_2_, probably owing to the instability of the surface Ti^3+^ species in air [39]. The Raman measurements support the negligible concentrations of both rutile TiO_2_ and HfTiO_4_. Moreover, in spite of the EDX data in the Raman spectra of all samples there are no bands of crystalline silica. Possibly, because of its high crystallization temperature (1350°C [40]), the SiO_2_ is amorphous. At the same time, it should be noted that all samples displayed the typical low-intensity D (1305 cm^–1^) and G (1590 cm^–1^) peaks (figure 5, inset) associated with the annealed carbon template in the amorphous and graphitized states [41].

**Figure 6.**
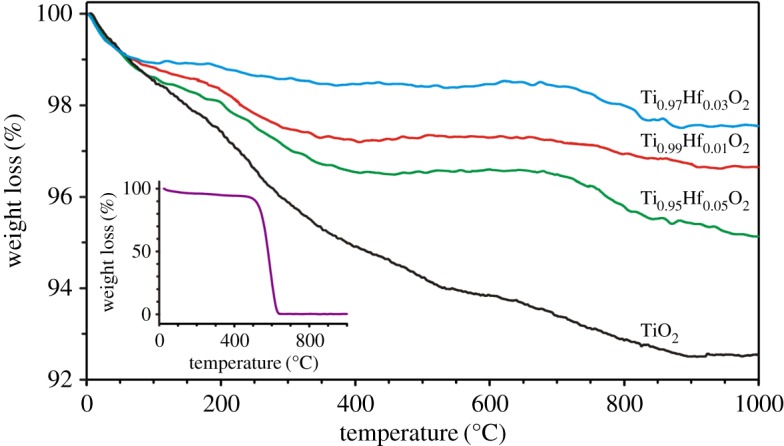
TGA curves for Ti_1–*x*_Hf*_x_*O_2_ samples with different Hf concentrations as well as for the Busofit-T055 carbon fibre template (inset).

To evaluate the concentration of carbon originated from the pyrolysis of Busofit-T055 fibre, thermogravimetric analysis (TGA) was performed. As shown in figure 6, the TGA curves of Ti_1–*x*_Hf*_x_*O_2_ (*x* = 0; 0.01; 0.03; 0.05) samples depict three steps of weight loss. The first region below 200°C is associated with the release of H_2_O adsorbed on the TiO_2_ surface [42]. The second interval from 200 to 650°C is related to the removal of residual carbon because the Busofit-T055 carbon fibre is fully decomposed at approximately 650°C (figure 6, inset). The third weight loss step between 650 and 850°C can be attributed to the dehydration of surface Ti(OH)_2_ [43]. Thus, the overall weight loss for all samples varies between 2.5% and 5.5%.

### 3.2. Electrochemical performance of Ti_1–x_Hf_x_O_2_

**Figure 7.**
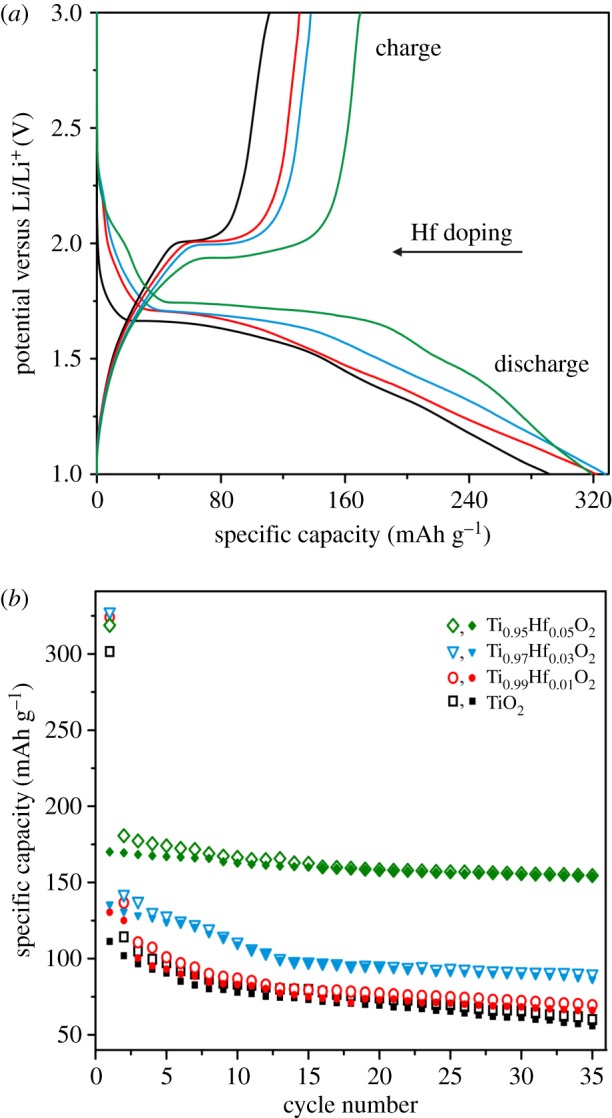
(*a*) First charge/discharge profiles and (*b*) cycling performances (open symbols, discharge; filled, charge) for Ti_1–*x*_Hf*_x_*O_2_ (*x* = 0.01; 0.03; 0.05) samples at *C*/10 in the voltage range of 1.0–3.0 V.

Figure 7*a* presents the charge/discharge profiles of undoped TiO_2_, Ti_0.99_Hf_0.01_O_2_, Ti_0.97_Hf_0.03_O_2_ and Ti_0.95_Hf_0.05_O_2_ for the first cycle. Note that initial specific capacity values for all samples are within the range of 300–325 mAh g^–1^. On the other hand, the theoretical capacity of TiO_2_ reaches 335 mAh g^–1^ (equation (3.1)). Possibly, lower first discharge capacities result from the presence of impurities (amorphous SiO_2_, HfTiO_4_). Additionally, limited Li^+^ ion diffusivity into TiO_2_ crystal structure is not ruled out. The first charge cycle characterizes the Li^+^ de-intercalation from TiO_2_-based samples. The reversible capacity of undoped TiO_2_ is equal to 111.3 mAh g^–1^ (insertion of 0.34 Li^+^ ions into the TiO_2_ structural unit). At the same time, Ti_0.99_Hf_0.01_O_2_, Ti_0.97_Hf_0.03_O_2_ and Ti_0.95_Hf_0.05_O_2_ samples yielded 130.5, 135.7 and 170.1 mAh g^–1^, respectively, which corresponds to de-intercalation of 0.39 Li^+^, 0.41 Li^+^ and 0.50 Li^+^ per titania unit. The obtained data represent the better Li^+^ ions pathways through Ti_0.95_Hf_0.05_O_2_ structure, which seem to be because of increase of the unit cell parameters after Hf^4+^ doping:

3.1 $$\text{Ti}{\text{O}}_{2}+x\text{L}{\text{i}}^{+}+x{\text{e}}^{-}↔\text{L}{\text{i}}_{x}\text{Ti}{\text{O}}_{2},\;0\leq x\leq 1.$$

After the 35th cycle (figure 7*b*), the reversible capacities stabilized at 55.9 mAh g^–1^ (TiO_2_), 66.1 mAh g^–1^ (Ti_0.99_Hf_0.01_O_2_), 86.4 mAh g^–1^ (Ti_0.97_Hf_0.03_O_2_) and 154.2 mAh g^–1^(Ti_0.95_Hf_0.05_O_2_). Thus, the Ti_0.95_Hf_0.05_O_2_ material shows higher stability during Li^+^ intercalation/de-intercalation into/from the crystal structure. The obtained results are in a good accordance with ones for nanostructured Zr-doped anatase TiO_2_ tubes published previously [28]. In particular, based on the Raman spectroscopy and charge/discharge tests, the effect of the dopant ionic radius on doped TiO_2_ electrochemical behaviour was established. At the same time, Ti_0.95_Zr_0.03_O_2_, which was the best sample, exhibited a reversible capacity of only 135 mAh g^–1^ after 35 cycles at a *C*/10 rate. Such worse performance can be explained by the slightly larger ionic radius of Zr^4+^ (0.72 Ǻ) when compared with that of Hf^4+^, which may cause the undesirable excessive lattice strain in anatase TiO_2_. This suggestion is also confirmed by our unsuccessful efforts to increase the Zr to Ti ratio to more than 0.03.

Overall, the capacity of nanostructured Hf-doped anatase TiO_2_ tubes is higher than that of Nb-doped TiO_2_ nanofibres (128 and 92 mAh g^–1^ for the 20th cycle at rates of *C*/20 and *C*/5, respectively), as demonstrated by Fehse *et al*. [44]. On the other hand, Wang *et al.* [32] reported an enhanced cycling performance (160 mAh g^–1^ after the 100 cycles at a *C*/6 rate) for mesoporous Nb-doped anatase titania with a specific surface of 128 m^2^ g^–1^. Hence, the relationship between the synthesis technique as well as morphology and electrochemical behaviour of the material is very strong. Note that, in the present work, the direct comparison of nanostructured undoped and Hf-doped anatase titania tubes reveals a superior cycling performance of the doped samples.

**Figure 8.**
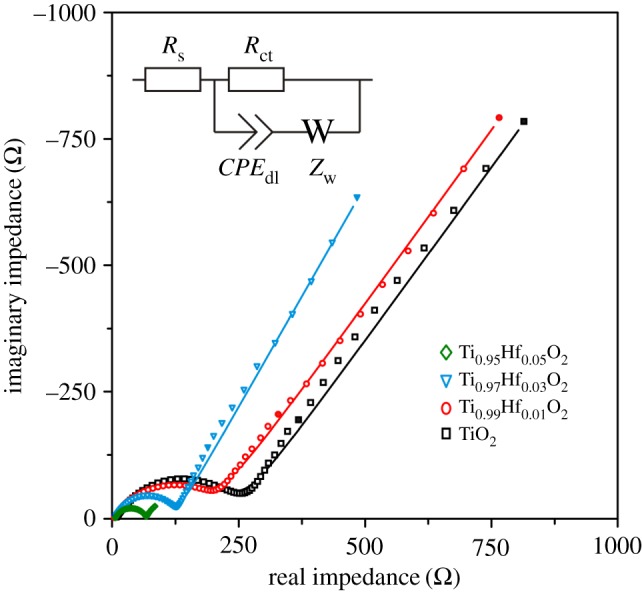
EIS spectra (the frequencies of 0.1 Hz and 1 Hz are marked by filled symbols) for undoped TiO_2_, Ti_0.99_Hf_0.01_O_2_, Ti_0.97_Hf_0.03_O_2_ and Ti_0.95_Hf_0.05_O_2_ electrodes fitted (solid lines) with equivalent circuits (inset).

To understand the reasons of higher performance behaviour of Hf-doped anatase TiO_2_ electrodes, the EIS method was applied. As shown in figure 8, the Nyquist plot consists of the high-frequency semicircle and the low-frequency arc. The EIS spectra have been fitted (table 2) using an equivalent circuit (figure 8, inset) composed of internal resistance *R*_s_, charge transfer resistance *R*_ct_ at the double layer *CPE*_dl_ (and, possibly, Li^+^ migration through the SEI) and Warburg impedance *Z*_w_ associated with diffusion of Li^+^ ions into the solid phase. The collected data demonstrate that the incorporation of Hf^4+^ ions into the anatase TiO_2_ crystal lattice enhances conductivity. Indeed, Ti_0.95_Hf_0.05_O_2_ presents the shorter diameter of a high-frequency semicircle, implying a smaller *R*_ct_ (60.9 *Ω*) in comparison with undoped TiO_2_ (249.2 *Ω*), Ti_0.99_Hf_0.01_O_2_ (204.4 *Ω*) and Ti_0.97_Hf_0.03_O_2_ (123.8 *Ω*). This shows that charge redistribution associated with Hf^4+^ doping is achieved by creation of oxygen vacancies in the anatase TiO_2_ lattice, and, possibly, owing to reduction of some Ti^4+^ ions to Ti^3+^ in order to maintain the charge balance.

**Table 2.** Calculated EIS parameters for Ti_1–*x*_Hf*_x_*O_2_ (*x* = 0.00; 0.01; 0.03; 0.05) electrodes.

sample	*R*_s_ (*Ω*)	*R*_ct_ (*Ω*)	*σ*_w_ (*Ω* s^–1/2^)	*D*_Li_ (cm^2^ s^–1^)
TiO_2_	6.2	249.2	516.8	7.3 × 10^–17^
Ti_0.99_Hf_0.01_O_2_	5.9	204.4	506.1	7.7 × 10^–17^
Ti_0.97_Hf_0.03_O_2_	6.1	123.8	342.1	1.7 × 10^–16^
Ti_0.95_Hf_0.05_O_2_	6.0	60.9	16.8	6.9 × 10^–14^

For further insight into improved cyclability of the Hf-doped anatase TiO_2_, low-frequency Warburg contribution in the Nyquist EIS spectra was analysed. The diffusion coefficients *D*_Li_ (cm^2^ s^–1^), calculated (table 2) from the Warburg factor *σ*_w_ (Ω s^–1/2^) equation (equation (3.2)), were 7.3 × 10^–17^ cm^2^ s^–1^ (TiO_2_), 7.7 × 10^–17^ cm^2^ s^–1^ (Ti_0.99_Hf_0.01_O_2_), 1.7 × 10^–16^ cm^2^ s^–1^ (Ti_0.97_Hf_0.03_O_2_) and 6.9 × 10^–14^ cm^2^ s^–1^ (Ti_0.95_Hf_0.05_O_2_). Hence, it is evident that Hf-doping improves Li^+^ diffusivity. The undoped TiO_2_ is characterized by almost three orders of magnitude slower solid-state diffusion of Li^+^ when compared with Ti_0.95_Hf_0.05_O_2_:

3.2 $$\sigma_{w}=RTS^{-1}n^{-2}F^{-2}{C_{\mathrm{Li}}}^{-1}{\left(2D_{\mathrm{Li}}\right)}^{-1/2},$$

where *R* is the gas constant (J mol^−1^ K^−1^), *T* is the temperature (K), *S* is the contact area between the electrode and electrolyte (cm^2^), *n* is the charge transfer number, *F* is the Faraday constant (C mol^−1^) and *C*_Li_ is the concentration of Li^+^ in TiO_2_ (mol cm^–3^). The Warburg factor was found (table 2) from the slope of the fitting line in the plot of the real axis impedance values *Z*′ versus the reciprocal square root of the angular frequencies *ω*^–1/2^ (electronic supplementary material, figure S3) according to the following relationship:

3.3 $$Z^{\prime }=R_{s}+R_{\mathrm{ct}}+\sigma_{w}\cdot \omega^{-1/2}.$$

**Figure 9.**
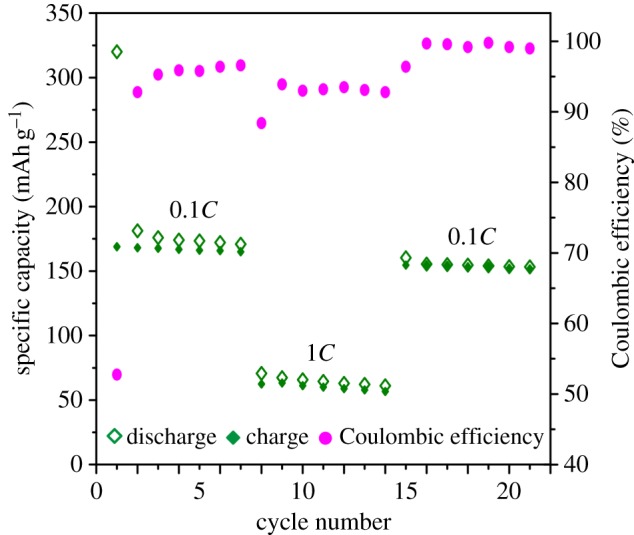
Specific capacity retention during Ti_0.95_Hf_0.05_O_2_ cycling at rates of *C*/10 and 1*C.*

Figure 9 reveals the reversibility of Li^+^ ion intercalation/de-intercalation into/from the Ti_0.95_Hf_0.05_O_2_ electrode after its cycling at different *C*-rates. Ti_0.95_Hf_0.05_O_2_ was first cycled at *C*/10 and, after seven cycles, the rate was increased up to 1*C*. A reversible capacity of 165.0 mAh g^–1^ was obtained at a *C*/10 rate after the seventh cycle. However, during the further charge/discharge cycling (8–14 cycles) at a rate of 1*C,* the capacity decreases down to 56.7 mAh g^–1^. At the same time, the specific capacity of Ti_0.95_Hf_0.05_O_2_ is restored up to about 151.8 mAh g^–1^ in the range from 15 to 21 cycles. The results show good durability of the Hf-doped anatase TiO_2_ electrode to the increased loading. The material shows that stable coulombic efficiency slightly depended on the current density.

## 4. Conclusion

In this work, a promising facile way of TiO_2_ modification by Hf-doping via a template sol–gel route in order to improve its Li^+^ storage properties is suggested. It was found that the as-synthesized Ti_1–*x*_Hf*_x_*O_2_ (*x* = 0.01; 0.03; 0.05) materials are microtubes (length of 10–100 µm, outer diameter of 1–5 µm) composed of nanoparticles with an average size of 15–20 nm. The surface areas of the Ti_1–*x*_Hf*_x_*O_2_ microtubes are in the range of 80–90 m^2^ g^–1^ and the pore volumes are 0.294–0.372 cm^3^ g^–1^. Results indicate that the Ti_0.99_Hf_0.01_O_2_, Ti_0.97_Hf_0.03_O_2_ and Ti_0.95_Hf_0.05_O_2_ materials have an anatase crystal structure. As demonstrated, the Hf^4+^ ions are homogeneously incorporated into the titania lattice by substitution of Ti^4+^. Owing to the incorporation of Hf ions the titania unit cell parameters are increased. When the Ti_1–*x*_Hf*_x_*O_2_ materials are used as LIB anodes, the lithiation and de-lithiation capacities as well as cycling performance of the as-synthesized TiO_2_-based materials significantly improved by Hf^4+^ doping. Indeed, 35-fold charge/discharge cycling at a *C*/10 rate in the range 1.0–3.0 V shows that the reversible capacity for Hf-doped TiO_2_ is significantly higher (e.g. 154.2 mAh g^–1^for Ti_0.95_Hf_0.05_O_2_) than that for the undoped titania (55.9 mAh g^–1^). The Hf^4+^ substitution of the Ti^4+^ in TiO_2_ structure leads to the charge transfer resistance *R*_ct_ decreasing (62.1 versus 280.1 *Ω* for Ti_0.95_Hf_0.05_O_2_ and undoped TiO_2_ respectively), which seems to be because of enhancement of conductivity. Moreover, the diffusion coefficient *D*_Li_ of Li^+^ ions is increased by almost three orders of magnitude from 7.33 × 10^–17^ cm^2^ s^–1^ (TiO_2_) up to 6.91 × 10^–14^ cm^2^ s^–1^ (Ti_0.95_Hf_0.05_O_2_). Overall, the better reversibility of the electrochemical process occurring in Hf-doped anatase TiO_2_ electrodes is associated with: (i) changing of the unit cell parameters, which facilitates Li^+^ ion diffusion; (ii) charge redistribution in the lattice, which improves conductivity of TiO_2_; and (iii) increased surface area, which enhances interfacial lithium storage. Comparison of the obtained results with those for Zr-doped anatase TiO_2_ published earlier demonstrates that ionic radius of dopant M*^n^*^+^ partially substituted with Ti^4+^ in the anatase TiO_2_ lattice is a key factor for the design of advanced anodes for high-safety LIBs.

## Supplementary Material

Supplementary Figures and Tables

## Data accessibility

Our data have been deposited in the Dryad Digital Repository: (http://dx.doi.org/10.5061/dryad.gf63r) [45].
