# Supplementary material for: Effect of Hf-doping on electrochemical performance of anatase TiO2 as an anode material for lithium storage
Source: R Soc Open Sci. 2018 Jun 6;5(6):171811. doi: 10.1098/rsos.171811 (PMC6030266; doi:10.1098/rsos.171811)
Supplement: Supplementary Figures and Tables [file rsos171811supp1.docx]

Electronic Supplementary Material

**Effect of Hf-doping on Electrochemical Performance of Anatase TiO_2_ as an Anode Material for Lithium Storage**

S. V. Gnedenkov*^a^*^,^*, S. L. Sinebryukhov*^a^*, V. V. Zheleznov*^a^*, D. P. Opra*^a^*, E. I. Voit*^a^*, E. B. Modin*^a,b,c^*, A. A. Sokolov*^a,b^*, A. Yu. Ustinov*^a^* and V. I. Sergienko*^a^*

*^a^* Institute of Chemistry, Far Eastern Branch of Russian Academy of Sciences, Vladivostok 690022, Russia.

*^b^* Far Eastern Federal University, Vladivostok 690950, Russia.

*^с^* National Research Centre “Kurchatov Institute”, Moscow 123182, Russia.

* Corresponding author: tel.: +7(423)2311889; fax: +7(423)2312590; e-mail: [ayacks@mail.ru](mailto:ayacks@mail.ru).


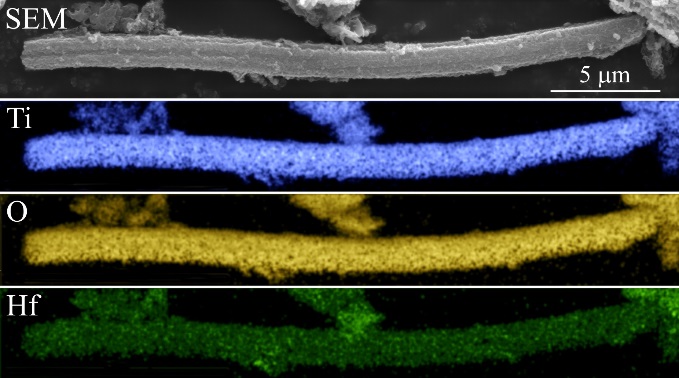


**Figure S1.** SEM image and elemental mapping of Ti, O, and Hf for Ti_0.95_Hf_0.05_O_2_

**
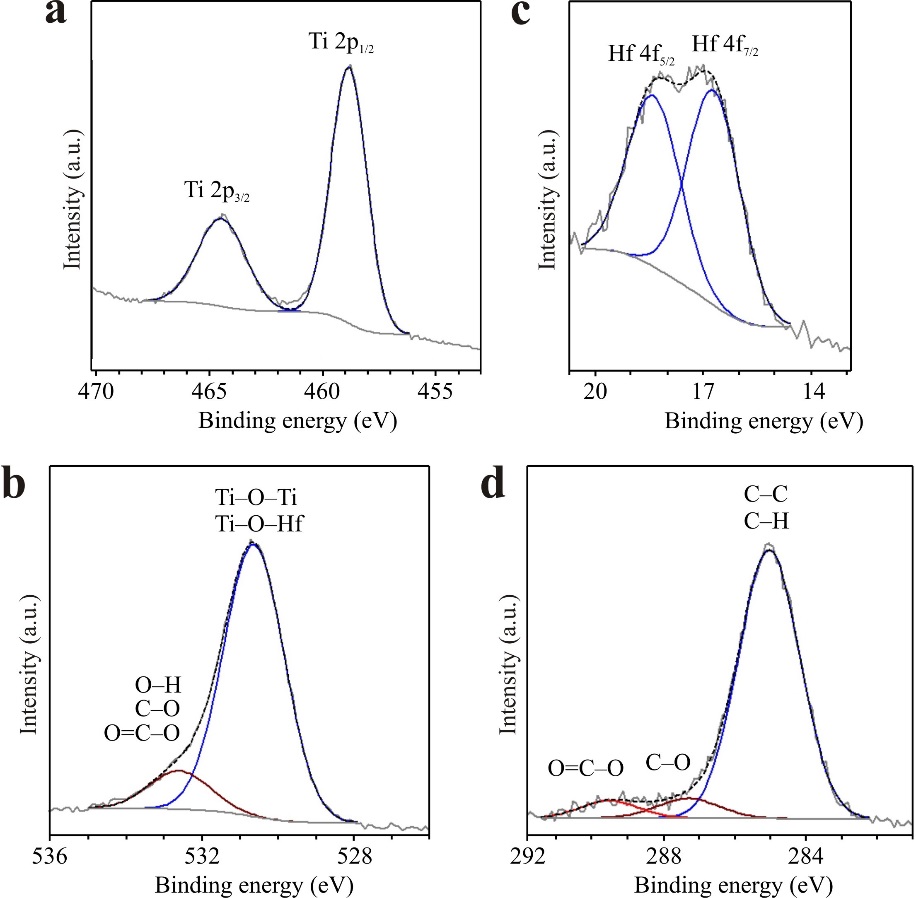
**

**Figure S2.** XPS high-resolution spectra of (a) Ti 2p, (b) O 1s, (c) Hf 4f, and (d) C 1s regions for Ti_0.95_Hf_0.05_O_2_ sample

**
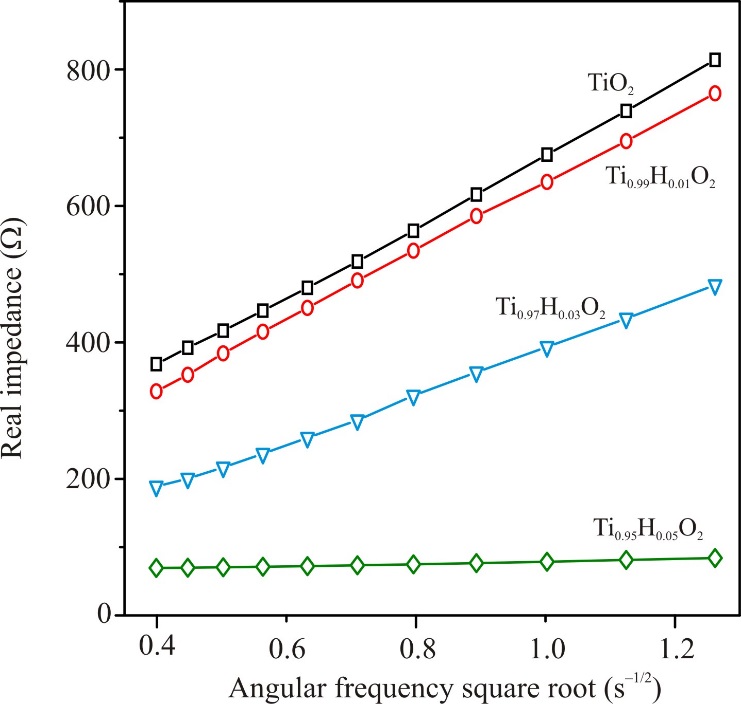
**

**Figure S3.** Dependence of *Z*′ on *ω*^–1/2^ at low frequencies

**Table S1.** Binding energy and atomic concentration of elements in Ti_0.95_Hf_0.05_O_2_ sample

| Peak | Concentration, at% | Binding energy, eV |
| --- | --- | --- |
| Ti 2p | 20.5 | 459.1 |
|  |  | 464.8 |
| О 1s | 46.0 | 530.6 |
|  | 6.3 | 532.5 |
| Hf 4f | 1.1 | 16.8 |
|  |  | 18.4 |
| C 1s | 1.3 | 289.5 |
|  | 1.8 | 287.3 |
|  | 23.0 | 285.0 |

**Table S2.** Dependence of *E_g_*_(1)_, *B*_1_*_g_*_(1)_, and *E_g_*_(3)_ peaks positions on Hf/Ti atomic ratio

| Hf/Ti  ratio | Peak maximum / cm^–1^ | | |
| --- | --- | --- | --- |
|  | *E_g_*_(1)_ | *B*_1_*_g_*_(1)_ | *E_g_*_(3)_ |
| 0.00 | 147.5 | 396.9 | 638.7 |
| 0.01 | 145.5 | 396.7 | 638.0 |
| 0.03 | 144.7 | 396.0 | 637.1 |
| 0.05 | 143.4 | 395.1 | 636.1 |
